# Assessment of common and differentially expressed proteins between diabetes mellitus and fatty liver disease: a network analysis 

**Published:** 2021

**Authors:** Kourosh Saki, Vahid Mansouri, Saeed Abdi, Mohammad Fathi, Zahra Razzaghi, Mehrdad Haghazali

**Affiliations:** 1 *Proteomics Research Center, Faculty of Paramedical Sciences, Shahid Beheshti University of Medical Sciences, Tehran, Iran*; 2 *Gastroenterology and Liver Diseases Research Center, Research Institute for Gastroenterology and Liver Diseases, Shahid Beheshti University of Medical Sciences, Tehran, Iran*; 3 *Critical Care Quality Improvement Research Center, Faculty of Paramedical Sciences, Shahid Beheshti University of Medical Sciences, Tehran, Iran*; 4 *Laser Application in Medical Sciences Research Center, Shahid Beheshti University of Medical Sciences, Tehran, Iran.*; 5 *Rajaie Cardiovascular Medical and Research Center, Iran University of Medical Sciences, Tehran, Iran*

**Keywords:** Nonalcoholic fatty liver disease, Diabetes mellitus, Network analysis, Bioinformatics, Biomarkers

## Abstract

**Aim:**

This study aimed to introduce the main biomarkers related to NFLD and diabetes II to determine common pathogenic and metabolite factors linking NFLD to diabetes II.

**Background:**

Nonalcoholic fatty liver disease (NFLD) is chronic hepatic failure with a broad range of hepatic disorders. NFLD and diabetes type 2 coexist regularly to drive adverse outcomes such as hepatocellular carcinoma and vascular complications

**Methods:**

The proteins related to NFDL and diabetes mellitus were extracted from String database. Proteins related to each disease were included in protein-protein interaction networks in Cytoscape software. Obtained networks were analyzed using Cytoscape network analyzer. The central nodes were determined as top hubs based on degree value. The top hubs related to NFLD and diabetes mellites were compared.

**Results:**

In total, 200 proteins related to NFDL and diabetes mellitus were found separately in String database and connected through undirected edges in individual networks. Central nodes based on degree value were determined for each disease. Ten percent of top nodes were selected based on degree value as the 20 top hubs for each disease. Target common hub proteins between NFDL and diabetes mellitus comprised INS, AKT1, ALB, PPARG, IL6, GPDPH, LEP, TNF, ADIPOQ, IGF1, TP53, MAPK3, and SIRT1.

**Conclusion:**

According to the results, 13 common and 14 discriminatory central dysregulated proteins were determined for NAFLD and diabetes mellitus.

## Introduction

 Diabetes mellitus type II and nonalcoholic fatty liver disease commonly coexist, regarding manifestation of metabolic syndrome (1), and each condition serves as a progression factor for the other (2). Fatty liver disease represents different ranges from simple steatosis and nonalcoholic steatosis to cirrhosis (3). The prevalence of fatty liver disease is 70% among diabetes II patients (4). Insulin resistance and obesity are linked to fatty liver disease (5). The staging of the disease is based on clinical parameters such as sex, age, liver function test, lipid profile, and blood sugar (6). Assessment and close monitoring of individuals for the presence of risk factors for both diseases is correlated with the recognition of biomarkers (7). Diagnosis of fatty liver disease according to variations and pathophysiologic alterations is still a matter of debate. Specific single markers related to hepatic pathophysiology, inflammation, and adipocytokines have attracted considerable attention; however, there is no single biomarker for the diagnosis and staging of fatty liver disease (8). Alanine aminotransferase (ALT) and aspartate aminotransferase (AST) are of clinical value and specifically used as hepatic damage indicators in clinics (9). C-reactive protein (CRP) of serum is another strong index predicting incidence of nonalcoholic fatty liver disease (10). Other research has introduced pentraxin as a diagnosis marker of liver fibrosis severity and differentiating between steatohepatitis and simple steatosis (11). It has been reported that there is a close relationship between diabetes mellitus and fatty liver disease. Research has revealed that an increased level of glycosylated hemoglobin (HbA1C) increases the risk of NFLD (12). There is much evidence about the molecular mechanism of diabetes mellitus and fatty liver disease based on bioinformatics findings and network analysis (13-16). As it has been reported, ANXA2, PRKCE, and OXT are critical genes related to fatty liver disease (17). Tousoulis et al. introduced TNF-α as a biomarker of diabetes mellitus (18). 

The present study aimed to identify the common biomarkers between diabetes mellitus and fatty liver disease through network analysis using the STRING database and Cytoscape software. 

**Figure 1:**
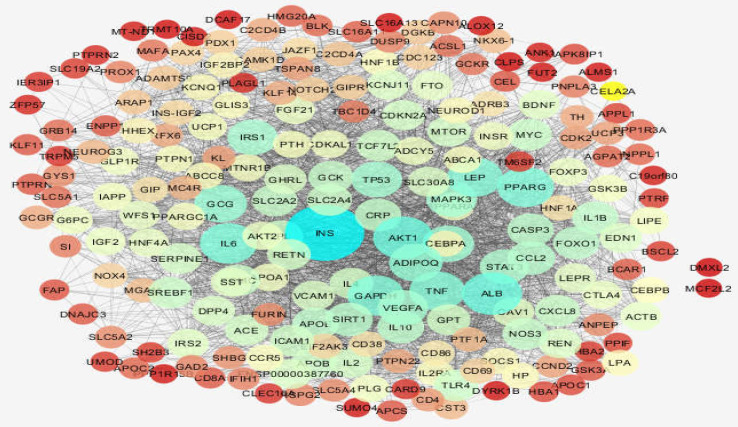
Diabetes mellitus network. The nodes are layout based on degree value

## Methods

A total of 200 genes related to the two diseases (diabetes mellitus and fatty liver disease) were retrieved from the STRING database (19) through several useful queries available in STRING, such as protein query and disease query. The STRING user can access dysregulated genes or proteins which are related to the disease under investigation through a disease query of the database. The genes were included in protein-protein network by Cytoscape software (20) for each disease separately. The constructed networks were analyzed by the Network Analyzer plugin of Cytoscape. The central nodes were determined as top nodes based on degree value, and the central nodes of the two analyzed networks were compared. 

## Results

**Figure 2:**
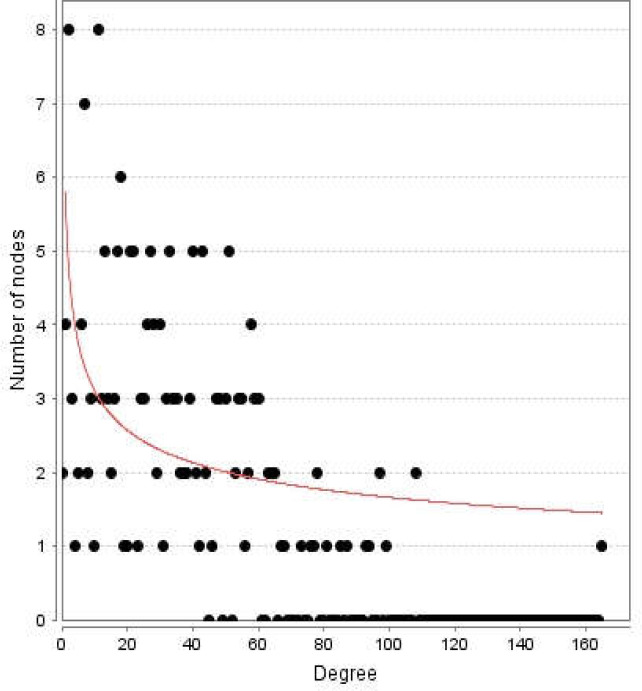
Degree distribution for the nodes of diabetes mellitus network

**Figure 3:**
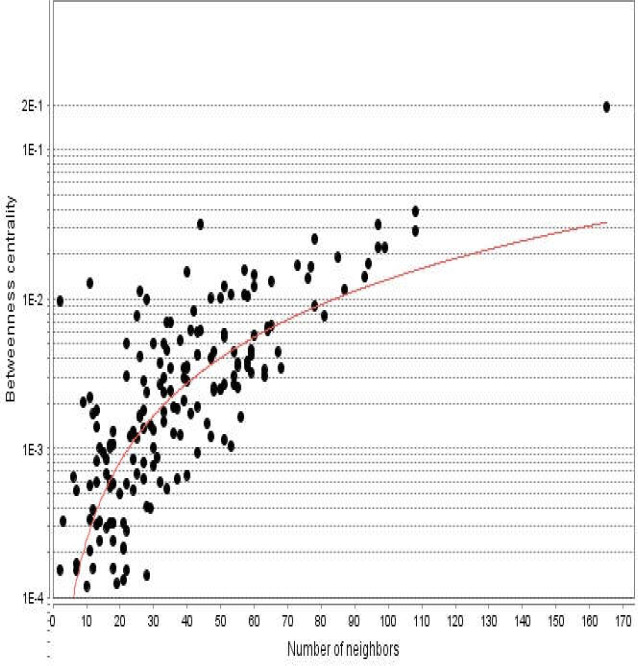
Betweenness centrality distribution for the nodes of diabetes mellitus network is presented. The vertical axis is represented in logarithm scale

Diabetes mellitus was searched in a disease query of STRING database. Two hundred proteins that were connected by 3516 edges appeared as an interactome. Except 2 proteins the other individuals were included in the main connected component. The network was analyzed and visualized based on degree value (Figure 1). The central nodes of the analyzed network were determined. Distribution of degree value and betweenness centrality are presented in Figures 2 and 3, respectively. 

**Figure 4:**
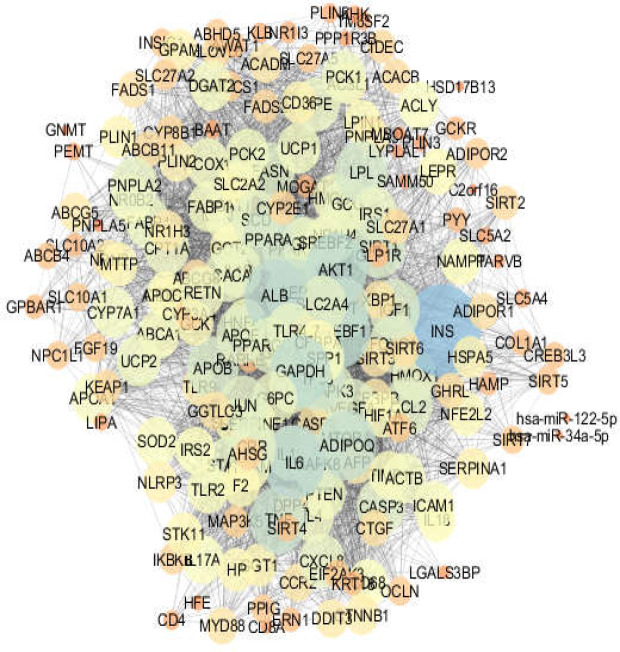
Fatty liver disease network. The nodes are layout based on degree value

Ten percent of top nodes, including 20 genes, based on degree value were identified as hub nodes (Table 1). A similar analysis was applied for fatty liver disease, the network of which included 200 nodes and 4672 edges (Figure 4). 

**Figure 5:**
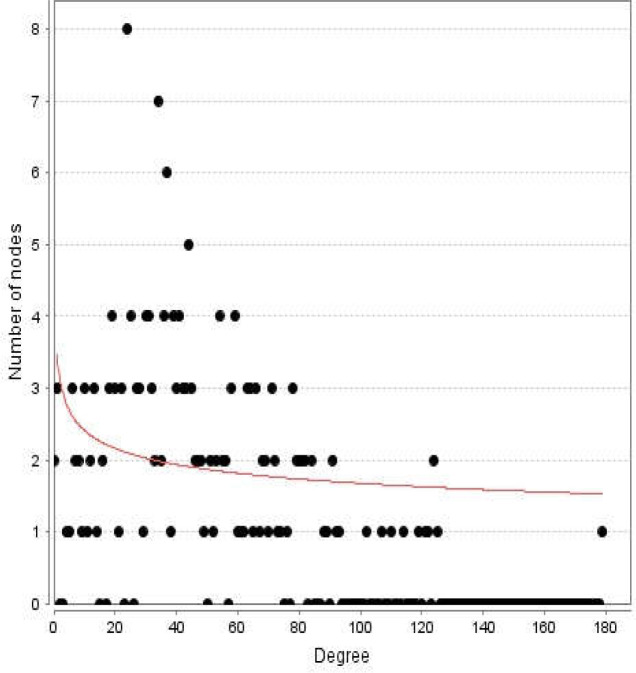
Degree distribution for the nodes of fatty liver disease network

**Table 1:** Hub nodes of diabetes mellitus network. The italic nodes are common between diabetes mellitus and fatty liver disease networks

R	Display name	Degree	Betweenness Centrality	Closeness Centrality	Stress
1	*INS*	162	1.000	0.836	51056
2	*AKT1*	106	0.128	0.666	14194
3	*ALB*	104	0.158	0.668	14772
4	*PPARG*	97	0.148	0.648	16486
5	*IL6*	95	0.076	0.644	10754
6	*GAPDH*	94	0.092	0.642	10798
7	*LEP*	91	0.051	0.630	8146
8	*TNF*	90	0.056	0.626	8958
9	*ADIPOQ*	86	0.041	0.620	6712
10	GCG	84	0.082	0.618	8714
11	*IGF1*	79	0.020	0.607	5334
12	*TP53*	77	0.112	0.607	10574
13	*MAPK3*	77	0.026	0.598	5662
14	IRS1	75	0.051	0.605	8612
15	VEGFA	74	0.066	0.598	7648
16	STAT3	71	0.061	0.585	7634
17	*SIRT1*	67	0.005	0.573	3190
18	CCL2	67	0.000	0.575	3312
19	CASP3	64	0.015	0.585	4790
20	APOE	64	0.015	0.582	4564

**Table 2:** Hub nodes of fatty liver disease network. The italic nodes are common between diabetes mellitus and fatty liver disease networks

R	Display name	Degree	BetweennessCentrality	ClosenessCentrality	Stress
1	*INS*	179	1.000	0.909	38236
2	*IL6*	125	0.168	0.716	15642
3	*AKT1*	124	0.104	0.711	12192
4	*PPARG*	124	0.168	0.721	13916
5	*ALB*	122	0.160	0.708	15030
6	SREBF1	121	0.208	0.713	15932
7	*GAPDH*	119	0.097	0.701	11688
8	*ADIPOQ*	114	0.112	0.696	10964
9	*LEP*	110	0.080	0.686	9864
10	*TNF*	107	0.088	0.670	11660
11	*TP53*	102	0.056	0.659	7774
12	LPL	93	0.056	0.648	8790
13	PPARA	92	0.072	0.640	8452
14	APOB	91	0.136	0.648	11470
15	*SIRT1*	91	0.024	0.636	5750
16	GPT	89	0.136	0.638	12180
17	*IGF1*	88	0.016	0.628	4718
18	*MAPK3*	84	0.008	0.620	4286
19	MAPK8	84	0.000	0.620	4354
20	FASN	82	0.024	0.624	6418

**Figure 6:**
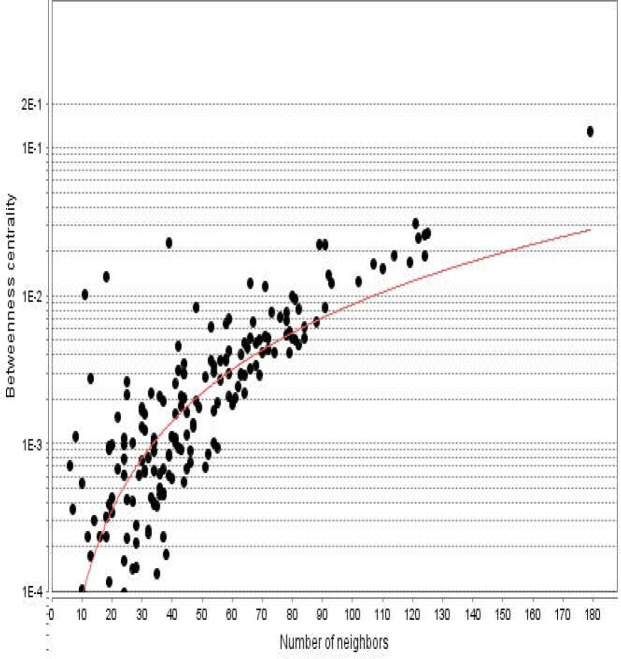
Betweenness centrality distribution for the nodes of fatty liver disease network. Vertical axis is represented in the logarithm scale

Distribution of degree value and betweenness centrality related to the fatty liver disease network are shown in Figures 5 and 6, respectively. As with analysis of the diabetes mellitus network, the central nodes of fatty liver disease were also determined. The 20 hub nodes of the fatty liver disease network are tabulated in Table 2.

## Discussion

As depicted in Tables 1 and 2, there are 13 common central proteins (those characterized with higher values of central parameters) (65% similarity) between diabetes mellitus and fatty liver disease: INS, IL6, AKT1, PPARG, ALB, GAPDH, ADIPOQ, LEP, TNF, TP53, SIRT1, IGF1, and MAPK3. 

As shown in Tables 1 and 2, most of the top central nodes of the two diseases are similar. As a result, 65% of central DEPs are common between the two studied diseases, which points to each disease being a risk factor for the other and emphasizes that these two diseases are closely related to each other (21). The prevalence of diabetes II is increased in NFLD and other chronic liver diseases (22). Neural and endocrine signaling between the liver and the brain in the pathophysiology of diabetes has been defined (23). A study revealed that when diabetes and obesity coexisted, 66% of the NFLD patients had advanced fibrosis (24). The differentially central nodes determined in this study can be considered as discriminate factors for diabetes mellitus and fatty liver disease. As depicted in Table 1, GCG, IRS1, VEGFA, STAT3, CCL2, CASP3, and APOE are the 7 DEPs that are related to diabetes mellitus as specific proteins but not seen among the central proteins of fatty liver disease. GCG, or the proglucagon gene, causes plasma glucose to rise in response to insulin-induced hypoglycemia and maintains the hyperglycemic condition in diabetes (25). Insulin receptor substrate 1 (IRS1) is critical in the insulin signaling pathway. IRS1 activates phosphatidylinositide kinase 3 (P13 kinase) as a second messenger to activate protein kinase BAKt to control and uptake glucose and glycogen synthesis through insulin activity (26, 27). Vascular endothelial growth factor A (VEGFA) expression is increased during diabetes to protect glomerular microvasculature and nephropathy inhibition (28). Signal transducer and activator of transcription 3 (STAT3) is involved in cytokine insulin resistance, but its role in muscle insulin resistance and diabetes is not completely clear (29). Monocyte chemoattractant protein 1 (CCL2) is a chief mediator of renin angiotensin system cytokines and emerges in the kidneys of diabetic neuropathy patients, leading to end stage renal failure (30, 31). Beta cell apoptosis plays a key role in the pathogenesis of diabetes through antigen cross-presentation mechanisms, and CASP3 is the major effector between caspases involved in beta cell apoptosis (32). The APOE gene (Apolipoprotein E) plays a role in lipid metabolism and brain physiology (33). The ɛ4 allelic variant of APOE could increase the risk for cognitive impairment and Alzheimer’s disease in diabetic patients (34, 35). 

On the other hand, based on data from Table 2, SREBF1, LPL, PPARA, APOB, GPT, MAPK8, and FASN are highlighted as specific DEPs which are related to fatty liver disease. SREBF1 is a key regulator of lipogenesis and insulin sensitivity, and it is associated with NFLD. Insulin affects hepatic lipogenesis mediated by SREBF1 gene expression (36). Activation of transcription factor LXRa increased SREBF1 expression, leading to hepatic lipogenesis and hypertriglyceridemia (37). SREBPs are involved in lipid and glucose hemostasis and play a key role in nonalcoholic steatohepatitis and obesity (38). LPL or lipoprotein lipase plays a central role in plasma lipids incorporation into tissue and regulates lipid metabolism. LPL expression is almost absent in adult livers (39). Peroxisome proliferator-activated receptors (PPARA) are involved in lipid and glucose transcriptional regulation metabolism, and their ligands have been introduced as possible traumatic agents for NFLD (40). APOB or apolipoprotein B is one of the largest proteins secreted by the liver in the form of VLDL (41). A genetic defect in APOB may lead to NFLD, as in familial hypobetalipoproteinemia (42). Elevated glutamate-pyruvate transaminase (GPT) values in fatty liver disease is significantly associated with diabetes and is a useful first indicator of glucose metabolism disturbance (43). MAPK8 or mitogen activated protein kinase 8 and other MAPKs participate in liver metabolism control. NFLD and other stress responses activate hepatic MAPKs to impair insulin action and lipid metabolism (44). Fatty acid synthase (FASN) catalysis the last step in fatty acid biosynthesis and could be a major determinant of the hepatic capacity to generate fatty acids in NFLD (45). The genes involved in diabetes and NFLD are INS, IL6, AKT1, PPARG, ALB, GAPDH, ADIPOQ, LEP, TNF, TP53, SIRT1, IGF1, and MAPK3, with 65% centrality regarding the current results. The presence of NFLD increases the incidence of diabetes II, while diabetes aggravates NFLD to more severe conditions such as steatohepatitis or cirrhosis (46). The INS gene has the highest degree value according to the current results, followed by IL6, AKT1, and PPARG, respectively. NFLD begins with the accumulation of triacylglycerol as cytoplasmic lipid droplets in 5% hepatocytes or triacylglycerol which can surge up to 95% in healthy people (47, 48). Lipid accumulation in the liver has been linked to the development of insulin resistance as a key feature of diabetes II (49). Primary insulin resistance in skeletal muscles leads to the distribution of substrates through the liver and simultaneous steatosis and accumulation of hepatic diacylglycerol with the activation of protein kinase C (50). Therefore, insulin is a key gene related to both NFLD and diabetes, as the current results have demonstrated. IL6 is a cytokine belonging to the interleukin family, and there are several polymorphisms for the IL6 gene associated with diseases such as diabetes, insulin resistance, and other metabolic syndromes (51).The human IL6a receptor gene maps in a region of chromosome 21 for replicate linkage to diabetes II (52). IL6 and inflammatory cytokines such as TNF-a play critical roles in the pathophysiology of human NFLD. IL6 is derived from many cells, including adipocytes, and serum levels of IL6 correlate with insulin resistance; however, adipose tissue-derived IL6 can regulate hepatic insulin resistance through SOCS3 upregulation (53). The current results were in accordance with the role of IL6 in both diabetes and NFLD. AKT1 is a serine/threonine protein kinase known as an important downstream target of the insulin signaling pathway with anti-apoptotic and peripheral metabolic effects (54). Metabolic syndrome is the clustering of metabolic abnormalities associated with increasing risk of diabetes II and cardiovascular disease. Researchers consider the role of AKT1 polymorphism as being associated with major metabolic syndrome components (55), which could be candidates for the incidence of diabetes and NFLD. The PPARG gene is expressed in liver hepatocytes, and its expression is correlated with fat accumulation in pathological conditions such as diabetes and NFLD (40). Transcriptional activation of PPARG induces fatty acid and lipid droplets accumulation in adipocytes, suggesting its preventative role against NFLD, although more investigations are required to coordinate with the current results (56). 

According to the current results, the similarity of the central dysregulated proteins between NAFLD and diabetes mellitus was determined to be 65%. GCG, IRS1, VEGFA, STAT3, CCL2, CASP3, and APOE as diabetes mellitus-related proteins and SREBF1, LPL, PPARA, APOB, GPT, MAPK8, and FASN as NAFLD-specific proteins were identified as possible discriminating factors between the two studied diseases. The current findings provide new information about the molecular features of the studied diseases, which can be useful in future supplementary investigations. More investigations are required to limit the number of introduced central proteins for clinical applications.

## Conflict of interests

The authors declare that they have no conflict of interest.
